# Chronic Monosodium Glutamate Administration Induced Hyperalgesia in Mice

**DOI:** 10.3390/nu10010001

**Published:** 2017-12-21

**Authors:** Anca Zanfirescu, Aurelia Nicoleta Cristea, George Mihai Nitulescu, Bruno Stefan Velescu, Daniela Gradinaru

**Affiliations:** Faculty of Pharmacy, “Carol Davila” University of Medicine and Pharmacy, TraianVuia 6, 020956 Bucharest, Romania; zanfirescuanca@yahoo.com (A.Z.); anicoletacristea@yahoo.com (A.N.C); bruno_velescu@yahoo.co.uk (B.S.V.); danielagrdnr@yahoo.com (D.G.)

**Keywords:** monosodium glutamate, hyperalgesia, hot-plate test, formalin test, nitric oxide synthase

## Abstract

Monosodium glutamate (MSG) is a widely used food additive. Although it is generally considered safe, some questions regarding the impact of its use on general health have arisen. Several reports correlate MSG consumption with a series of unwanted reactions, including headaches and mechanical sensitivity in pericranial muscles. Endogenous glutamate plays a significant role in nociceptive processing, this neurotransmitter being associated with hyperalgesia and central sensitization. One of the mechanisms underlying these phenomena is the stimulation of Ca^2+^/calmodulin sensitive nitric oxide synthase, and a subsequent increase in nitric oxide production. This molecule is a key player in nociceptive processing, with implications in acute and chronic pain states. Our purpose was to investigate the effect of this food additive on the nociceptive threshold when given orally to mice. Hot-plate and formalin tests were used to assess nociceptive behaviour. We also tried to determine if a correlation between chronic administration of MSG and variations in central nitric oxide (NO) concentration could be established. We found that a dose of 300 mg/kg MSG given for 21 days reduces the pain threshold and is associated with a significant increase in brain NO level. The implications of these findings on food additive-drug interaction, and on pain perception in healthy humans, as well as in those suffering from affections involving chronic pain, are still to be investigated.

## 1. Introduction

Monosodium glutamate (MSG), the salt of glutamic acid, is widely used as a food additive (E621). Two enantiomers are known, but only the naturally occurring isomer *L* is used as a flavor enhancer. Kwok, in 1968 [1], reported transient subjective symptoms (flushing, headache, numbness, general weakness, palpitation) following consumption of Chinese dishes known to contain high concentrations of E621. Several human studies were conducted afterwards to determine if a causal relationship existed between MSG and this symptom complex, but the results were inconsistent. The Joint FAO/WHO Expert Committee on Food Additives in 1971 [2], 1974 [3], and 1987 allocated it an “acceptable daily intake (ADI) not specified”, considering MSG consumption to be safe [4]. The total intake of glutamate from food in European countries was assessed to range between 5 and 12 g/day, taking into account both natural and added glutamate [5].

Although MSG consumption is believed to be safe, several reports correlate MSG consumption with a series of unwanted reactions, including headache and mechanical sensitivity in pericranial muscles [5,6]. Clinical reports state that MSG consumption increases the frequency of fibromyalgia symptoms [7].

l-glutamate is a fast excitatory neurotransmitter with a significant role in nociceptive processing [8]. Two types of glutamate receptors are currently known: ligand-gated ion channels (NMDA, AMPA, kainate), and G protein-coupled receptors (metabotropic receptors) [9]. These receptors are well expressed in the central and peripheral nervous system, and have a high distribution in pain pathways [10,11,12]. Intraperitoneal or intrathecal administration of glutamate or agonists selective for one type of glutamate receptor induces nociceptive behaviors. Treatments with NMDA and AMPA antagonists or with inhibitors of glutamate release significantly reduce the hyperalgesia induced in experimental rodent models of acute inflammatory and neuropathic pain [13,14].

One of the mechanisms linked with NMDA-mediated hyperalgesia is stimulation of Ca^2+^/calmodulin sensitive nitric oxide synthase, and a subsequent increase in nitric oxide (NO) production [15]. This molecule is a key player in nociceptive processing, with implications in acute [16] and chronic pain states [13]. The peripheral and central (mostly spinal) role of NO in nociceptive response was investigated in different animal models. Rat response to mechanical stimuli in a paradoxical sleep deprivation hyperalgesia model has been associated to nitric oxide synthase (NOS) activity enhancement in dorsolateral grey matter, leading to changes in the descendent modulating pain pathways [17]. Knock-out mice, lacking NOS encoding genes, showed a decrease of the tactile allodyniain mechanical stimulus test [18]. Nx-nitro-l-arginine methyl ester (l-NAME), a non-selective NOS inhibitor reduced the behavioral signs of neuropathic pain induced in rats by constricting the spinal [19] and sciatic [20] nerves. Intrathecal administration of l-NAME or of methylene blue, a soluble guanylatecyclase inhibitor, suppresses the thermal hyperalgesia induced in the sciatic nerve constriction model. Pretreatment with NOS inhibitors significantly attenuated the thermal hyperalgesia induced by the intraplantar injection of complete Freund’s adjuvant in mice [21].

Taking into account the involvement of endogenous glutamate in pain processing and the different existing reports on MSG, we hypothesized that oral administration of this flavor enhancer would modify the nociceptive threshold when orally administered in mice. We also tried to determine some of the molecular changes underlying this effect.

## 2. Materials and Methods

### 2.1. Chemicals

Drugs and reagents employed were as follows: l-glutamic acid monosodium salt monohydrate, l-arginine, formaldehyde solution for molecular biology (36.5–38% in water), phosphate-buffered saline (PBS), Folin & Ciocalteu’s phenol reagent, *N*-(1-naphthyl)-ethylenediaminedihydrochloride, cadmium chloride. All reagents were purchased from Sigma-Aldrich (St. Louis, MO, USA).

### 2.2. Animals

All experiments were performed in 5 weeks male NMRI mice (*n* = 60; 30 ± 3.6 g), purchased from UMF Biobase (Bucharest, Romania). They were housed 10 animals per cage (35.5 cm × 22.9 cm × 15.2 cm), in a ventilated cage system, with a bedding of wood sawdust, under controlled light/dark cycle conditions (12 h light/12 h dark; lights on at 6:00 a.m.), with free access to water and food pellets. The temperature ranged between 20 and 22 °C, and the relative humidity was maintained at 35–45%. All reagents were purchased from Sigma-Aldrich (St. Louis, MO, USA). All procedures were carried out according to EU Directive 2010/63/UE, and with the approval of the Institutional Animal Care and Use Committee. The study was approved by the Bioethics Commission of the University of Medicine and Pharmacy Bucharest with the ethical approval code 589/04.09.2016. For each experiment we used 30 mice, divided in three equal groups. The animals received the test solutions for 21 days by means of a straight animal ball-tipped feeding needle. The body weight evolution was constant during the experiment for all three groups, exhibiting no significant differences. After 21 days the mice body weight was 41 ± 4 g.

### 2.3. Formalin-Induced Nociception

Group I represented the control, and was given distilled water 1 mL/100 g, group II received 150 mg/kg MSG (1.5% MSG solution), and group III 300 mg/kg MSG (3.0% MSG solution). All solutions were prepared in distilled water. The doses of MSG used were selected following multiple tests, as reported previously [22]. Mice were injected with 20 μL of formalin reagent containing formaldehyde 1.2% in PBS into the plantar surface of the left hind-paw with a 30-gauge needle. They were then placed in a Plexiglas box (30 cm × 30 cm × 30 cm) with a mirror below the floor at a 45 degree angle to allow an unobstructed view of the paws. The total time spent by animal either licking or biting the injected paw (reaction time) was recorded for the following 50 min. We took into account the biphasic behavior induced by formalin: an initial acute phase (with a duration of 0–5 min, neurogenic pain), followed by a prolonged tonic response (between 25 and 50 min, inflammatory pain). Between phases 1 and 2, there was an intermittent period where little nociceptive behavior was observed. The total time spent by animal licking or biting the paw injected with formalin was considered an index of nociception in the formalin test.

### 2.4. Hot-Plate Latency Assay

Groups of ten mice received doses of 150 and 300 mg/kg MSG orally for 21 days, while the control group was administered with the vehicle. On day 21, animals were treated and brought into the testing room one hour before testing. Animals were placed in the testing chamber and allowed to acclimate for 1 h. For testing, mice were put into a fiberglass cylinder (15 cm diameter, 30 cm high) on a metal base, maintained at a temperature of 53 ± 1 °C. Paw withdrawal latency to thermal noxious stimuli and latency of jumping response were used to assess the effects of substances on the thermal nociceptive threshold (Hot Plate, UgoBasile, Italy). In the absence of a response, the cut-off time was set to 45 s to prevent extensive damage of tissue. Hind-paw lifting was defined as lifting a hind-paw completely off the hot-plate. After 2 h, animals were sacrificed by decapitation, and the brain was harvested on ice. The biological material was used for the biochemical assays.

### 2.5. Assay of NO-Synthase (NOS) Activity

Total NOS activity was determined in crude tissue homogenates using the Griess method for human plasma adapted for brain tissue [23]. Using this method, we determined the concentration of NO metabolites: nitrites and nitrates (NOx). Freshly harvested brains were homogenized with PBS 1:5, centrifuged 10× *g* min/2000 rpm and the supernatant further used. The brains were homogenized, centrifuged and then kept on ice until all culls were complete. Cofactors and substrate were added according to Table 1.

The reagents were stored on ice. Between each addition of cofactor/substrate, the aliquots were vortexed. The cofactors/substrates were prepared in PBS. When left for 60 or 30 min, the aliquots were left on a rocker. The nitrates were reduced to nitrites in the presence of cadmium, and treated with a diazotizing reagent, sulfanilamide, in acidic media to form a transient diazonium salt. This intermediate reacts with the coupling reagent, *N*-(1-naphthyl)-ethylenediaminedihydrochloride, to form a stable azo compound. The intense purple color of the product allows nitrite assay with high sensitivity, which was used to measure nitrite concentration spectrophotometrically at 660 nm (Chemwell2010, Awareness Technology, Inc., Palm City, FL, USA).

### 2.6. Total Protein Assay

The total protein concentration for the same samples of brain tissue was determined using the Lowry protein assay [24]. Diluted protein solutions treated with copper salts in basic pH and Folin & Ciocalteu’s phenol reagent (hexavalent phosphomolybdic/phosphotungstic acid complexes) lead to the formation of blue compounds, whose concentration is linearly proportional to the protein concentration in the sample. NOx concentration was expressed for 1mg of protein.

### 2.7. Statistical Analysis

Statistical analysis was performed using GraphPad Prism version 5.00 for Windows, (GraphPad Software, San Diego, CA, USA). The type of distribution of the animal response was established with D’Agostino & Pearson test. Data are reported as means ± standard error of the mean (SEM), and were analyzed statistically using parametrical Student’s *t*-Test, a confidence interval (CI) of 90%, and with *p* values of 0.05 or less being considered to be significant.

## 3. Results

### 3.1. Formalin-Induced Nociception

**Figure 1:**
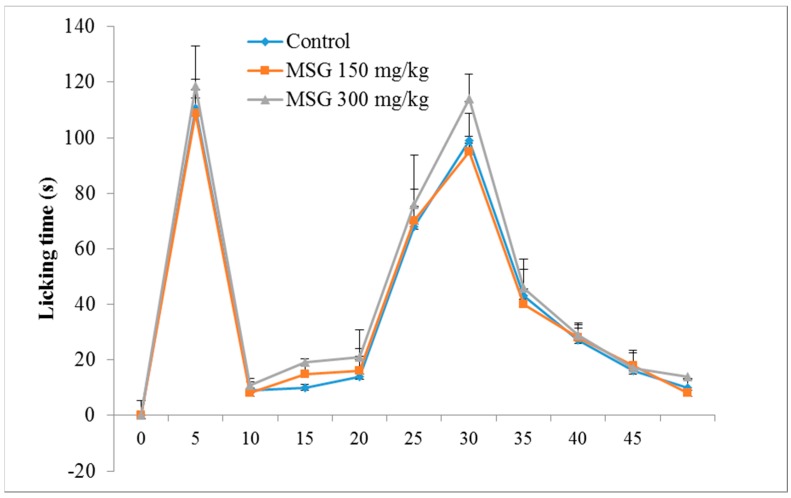
Time course of the formalin test in control (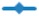) and in Monosodium glutamate (MSG) treated mice (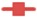 MSG 150mg/kg; 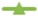 MSG 300 mg/kg). Values represent means ± standard error of the mean (SEM) of 10 animals.

The control group exhibited a typical biphasic nociceptive response: increased time of reaction in the first 5 min, a reduction in response for approximately 10 min, and a subsequent increased level of nociceptive response, which began 15 min after formalin injection, and lasted until the end of the experiment. The results (Figure 1) showed that, in the first phase of the formalin test, there were no significant differences between the tested groups, although a slight increase in reaction time (7.05%) was noticed for group III-MSG 300 mg/kg vs. control.

For phase 2, characterized by inflammatory pain, MSG administered in a dose of 300 mg/kg determined a significant increase in the mean reaction time vs. the control group (11.31%, *p* < 0.05). In addition, the frequency of nociceptive responses during the normally quiet intermediate phase was similar to that of the late phase of the formalin test.

### 3.2. Hot-Plate Latency Assay

**Figure 2:**
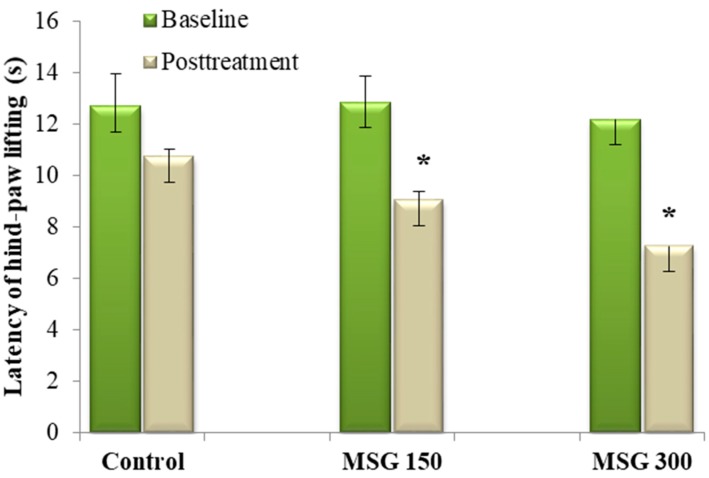
Medium latency of hind-paw lifting ± SEM before and after 21 days of MSG exposure. * Statistical significance vs. baseline (Student’s *t*-Test, 90% CI, *p* < 0.05) and control group (Student’s *t*-Test, 90% CI, *p* < 0.05).

We tested the effects of oral dosing of MSG on baseline heat pain thresholds in mice. Previous studies showed no significant changes vs. the control group in hot-plate assay after seven days of MSG oral administration, for either 150 mg/kg or 300 mg/kg dosage. Only a slight, non-significant, reduction of the pain threshold was seen, after 14 days of MSG 300 mg/kg administration (data not published). Oral administration of MSG in doses of 150 mg/kg and 300 mg/kg significantly reduced the average latency of hind-paw lifting vs. baseline (−29.69% and −40.43%, respectively, all *p* < 0.05), following 21 days of administration. However, only the dose of 300 mg/kg MSG significantly reduced (−30.03%, *p* < 0.05) the average latency of hind-paw lifting vs. control (Figure 2). For the latency of jumping time, a significantly larger number of animals jumped when compared to the control group (data not shown).

### 3.3. Assay of NO-Synthase (NOS) Activity

**Figure 3:**
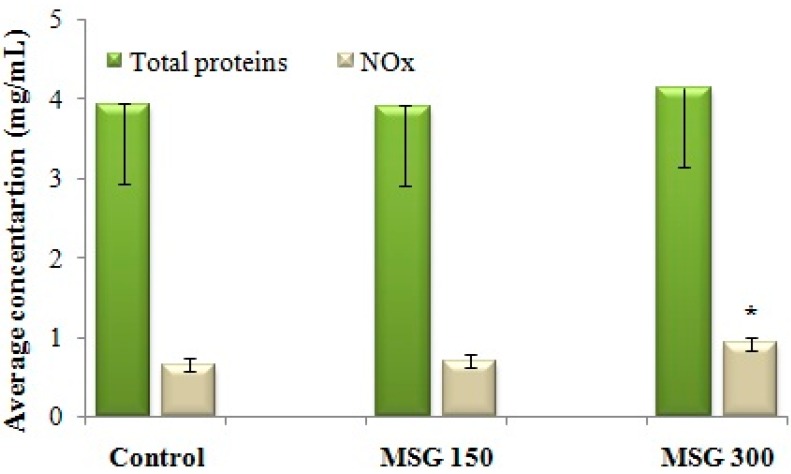
Average concentration of NO metabolites (NOx) in brain tissue after 21 days of exposure to distilled water (control) or MSG (150 mg/kg or 300 mg/kg). NOx concentration, as determined with the Griess method (x), is reported against the total protein concentration assessed in the same sample of brain tissue (y). The NOx concentration per 1 mg of protein = x/y. * Statistical significance vs. control (Student’s *t*-Test, 90% CI, *p* < 0.05).

NOS activity was assessed indirectly, by determining the concentration of NO metabolites (NOx). Administration of 300 mg/kg MSG for 21 days determined an increase of NOx concentration in the brain, and therefore an intensification of NOS activity in brain tissue (Figure 3). There were no changes in the total concentration of brain proteins when compared with the control.

We established a direct correlation between the increase in NOx concentration and the hyperalgic response to repeated administration of MSG (Spearman correlation, *p* < 0.05). Further studies will establish the effect of MSG administration on NOx in nociceptive behavior-naïve mice.

## 4. Discussion

Administration of MSG in a dosage of 300 mg/kg reduces the thermal nociceptive threshold and increases nociceptive behavior. The effect of MSG on the thermal threshold was correlated with an increase in NOx concentration, which suggests that the hyperalgic effect of MSG could be mediated via this messenger.

There is no literature data on the effect of MSG oral administration on the thermal threshold, as far as we know. Hot-plate is an objective, quantifiable, specific central antinociceptive test used to study the response to a noxious thermal stimulus [25,26]. The involvement of nitric oxide in nociceptive processing is generally recognized, with this molecule playing a pivotal role in hyperalgesia and central sensitization [27].

The formalin test assesses nociception as well as inflammation. Injection of formalin induces a biphasic response; the early first phase is neurogenic, and results from direct stimulation of nociceptors. This leads to the activation of sensory C-fibers through the transient receptor potential A1 receptors [28] in the paw, and the consequent release of substance P and bradykinin [29]. The late phase is inflammatory, and is due to the release of histamine, serotonin, bradykinin, prostaglandins, sympathomimetic amines, tumor necrosis factor-alpha and interleukins [30,31]. Central sensitization is partly responsible for the prolonged second phase of this test [32].

MSG administered in a dose of 300 mg/kg determined a significant increase in the mean reaction time vs. the control group for phase 2 of the formalin test. More importantly, a similar pattern of nociceptive response was seen during the normally quiet intermediate phase, as in the late phase of the formalin test. This may suggest MSG treatment shifts the tonic phase of formalin nociception to an earlier time point, possibly by enhancing the processes involved in mediating the sensitization in the spinal cord. Data from the literature support our findings regarding the correlation between the administration of MSG and the results of the formalin test. The enhancement of hyperalgesia in rats treated with l-glutamate was reversed by pretreatment with l-NAME. l-glutamate enhances hyperalgesia and persistent nociception following formalin-induced tissue injury, which seems to be mediated by intracellular messengers, including nitric oxide. Intrathecal pretreatment with inhibitors of nitric oxide synthase reduced formalin injury-induced nociceptive behaviors. l-NAME affected the tonic, but not the acute, phase of the formalin response. Conversely, formalin-induced nociceptive responses were enhanced by stimulators of nitric oxide such as sodium nitroprusside [33].

The increase in response during the inflammatory phase could be explained by a NO-induced rise in peripheral concentrations of prostaglandin E2 and prostacyclin, a phenomenon reported by several authors [13,16]. This increase was completely impeded by the NOS inhibitors. In vitro assays support the hypothesis that NO activates cyclooxygenases [34]. These results indicate that NO can also induce peripheral hyperalgesia by regulation of the expression and/or activity of cyclooxygenases, resulting in an increase of prostaglandins release.

The implications of MSG consumption could go further than hyperalgesia, since glutamate induces astrocyte mitochondrial apoptosis. Glutamate increased the expression of representative apoptotic markers, including cleaved caspase-8, cleaved caspase-9, and cleaved caspase-3, as well as level key markers in endoplasmic reticulum stress, in primary cultured spinal cord astrocytes [35]. This opens the way for further investigations: Does administration of MSG impacts spinal cord astrocytes? And what would the consequence be, taking into account the multiple roles of these cells in central nervous system CNS, and given that their apoptosis subsequently leads to CNS injury?

Our findings raise some questions regarding the safety of long-term MSG consumption, and represent a good starting point for any clinical tests focused on examining the impact of MSG nutritional intake on hyperalgesia.

One of these questions concerns the possible interactions between MSG and some analgesics; opioids, non-steroidal anti-inflammatory drugs, and natural products have peripheral and central antinociceptive effect mediated via l-arginine/NO-cGMP pathway [36,37]. Ventura-Martinez et al. showed that acute or chronic administration of l-arginine to mice decreases morphine analgesia. The inhibitory effect of l-arginine on morphine-analgesia and entry into the CNS is blocked by the NOS inhibitor l-NNA [38].

Another issue regards the effect of MSG consumption on pain perception in healthy humans, as well as in those suffering from maladies involving chronic pain. Already, this food additive is classified as a causative substance of headache in the International Classification of Headache Disorders, 3rd edition (ICHD-III beta), and has been found to increase the frequency of fibromyalgia symptoms [7]. Further studies need to be made in order to evaluate the impact of MSG consumption on pain associated with injuries, nerve lesions and degenerative diseases such as osteoarthritis.

## Figures and Tables

**Table 1:** Working method for NO metabolites (NOx) determination.

Reagents (μL)	Test	Control
Supernatant	360	360
PBS	310	540
l-Arginine ^1^	60	-
FAD ^2^	60	-
NADPH ^3^	10	-
37 °C, 60 min		
Cadmium chloride	0.5 g/mL	0.5 g/mL
25 °C, 30 min		
Griess reagent	1000	1000

^1^ 1.3 mg l-arginine/10 mL solution; ^2^ 0.6 mg FAD/10 mL solution; ^3^ 12.5 mg NADPH/1 mL solution. PBS, phosphate-buffered saline.
